# Severe Mitral Stenosis and Regurgitation Due to Bioprosthetic Valve Failure with Massive Pannus

**DOI:** 10.70352/scrj.cr.24-0095

**Published:** 2025-04-16

**Authors:** Ryota Hara, Sho Torii, Joji Ito, Yohei Ohno, Minoru Tabata

**Affiliations:** 1 Department of Cardiovascular Surgery, Tokyo Bay Urayasu Ichikawa Medical Center, Urayasu, Chiba, Japan; 2 Department of Cardiology, Tokai University School of Medicine, Isehara, Kanagawa, Japan; 3 Department of Cardiovascular Surgery, Juntendo University Graduate School of Medicine, Tokyo, Japan

**Keywords:** bioprosthesis, echocardiography, heart failure, redo mitral valve replacement, pannus

## Abstract

**INTRODUCTION:**

Bioprosthetic valve failure after mitral valve replacement is a well-recognized phenomenon, with pannus overgrowth being one notable cause. The standard treatments include redo surgical mitral valve replacement and the less invasive transcatheter treatment, mitral valve-in-valve. However, the feasibility and safety of performing mitral valve-in-valve when pannus overgrowth has reduced the valvular opening to a mere pinhole remains uncertain.

**CASE PRESENTATION:**

A 76-year-old woman, who had previously undergone mitral valve replacement, was admitted for congestive heart failure. Severe mitral stenosis and severe mitral regurgitation were diagnosed using transthoracic echocardiography. During redo mitral valve replacement, we observed that the prosthetic valve leaflets on the left atrial side were almost entirely covered with pannus tissue, leaving only a central pinhole for blood flow. Macroscopic and microscopic examination of the bioprosthesis revealed accordion-like leaflet deformation on the ventricular side.

**CONCLUSIONS:**

Although the transcatheter valve-in-valve procedure is recognized as a less invasive treatment option for degenerated biological valves, in certain cases such as ours, open surgery becomes imperative as the most appropriate treatment.

## Abbreviations

IABP
: intra-aortic balloon pumping
MS
: mitral stenosis
MR
: mitral regurgitation
MVR
: mitral valve replacement
MVIV
: mitral valve-in-valve
SVD
: structural valve degeneration

## INTRODUCTION

Bioprosthetic valve failure after mitral valve replacement (MVR) is a well-documented phenomenon, with pannus overgrowth being one notable cause.^1)^ The standard treatments include redo surgical MVR and the less invasive transcatheter treatment, known as mitral valve-in-valve (MVIV).^2)^ MVIV has gained increasing attention owing to its minimal invasiveness. However, the feasibility and safety of performing MVIV when pannus overgrowth has reduced the valvular opening to a mere pinhole remains uncertain. Since MVIV is not yet approved in Japan, clinically significant bioprosthetic valve failure is typically managed through redo surgical MVR. This report discusses a case managed under such circumstances, including the intraoperative findings and pathological evaluation, highlighting the challenges and considerations involved in treating bioprosthetic valve failure with extensive pannus formation.

## CASE PRESENTATION

**Fig. 1:**
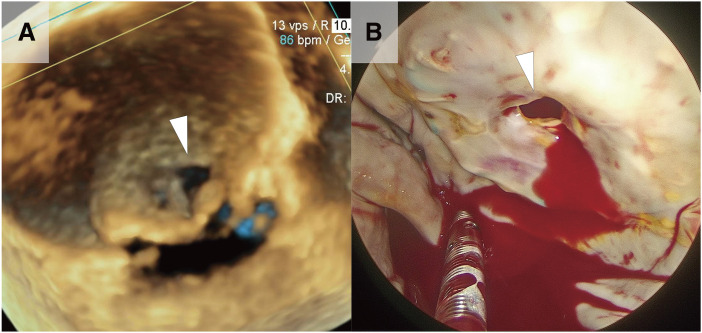
Preoperative and intraoperative images of stenotic bioprosthesis. (**A**) 3D image of the mitral bioprosthetic valve. (**B**) Intraoperative view (the white arrows indicate the restricted opening of the prosthesis).

A 76-year-old woman underwent MVR with a 29-mm Mosaic porcine valve (Medtronic, Minneapolis, MN, USA) and pledgeted everting mattress sutures, tricuspid band annuloplasty, and coronary artery bypass grafting 7 years before. She was admitted to our hospital for cardiogenic shock and congestive heart failure, which required mechanical ventilation and intra-aortic balloon pumping (IABP). Transthoracic echocardiography showed severe mitral stenosis (MS) with a mitral valve area of 0.5 cm^2^, a mean pressure gradient of 9 mmHg, and severe mitral regurgitation (MR). Transesophageal echocardiography showed severely restricted opening of the prosthesis with a straight and wide regurgitant jet from its center. This restricted opening was primarily due to massive pannus formation, which covered the prosthetic valve leaflets, leaving only a pinhole-sized passage at the center for blood flow (**Fig. 1A**). Although heart failure and cardiogenic shock were stabilized with mechanical support and medical management, the patient remained dependent on IABP and mechanical ventilation after 2 weeks. Therefore, we decided to proceed with redo MVR via a re-sternotomy.

**Fig. 2:**
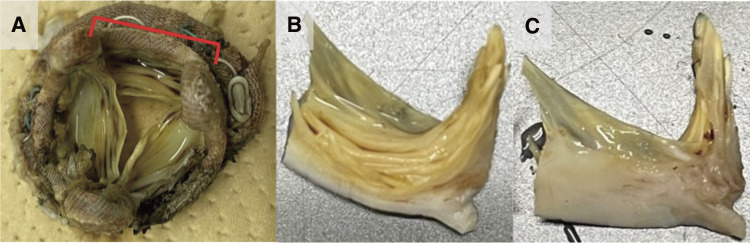
Removed mitral bioprosthetic valve. (**A**) Overview; the section marked in red is cut into (**B**) and (**C**). (**B**) Ventricular side; (**C**) atrial side.

**Fig. 3:**
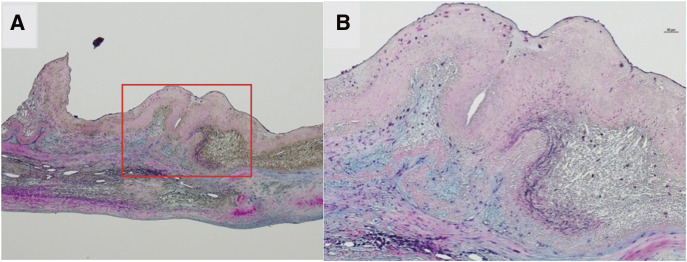
Microscopic sections of biological valves, 4×, Movat Pentachrome stain. (**A**) Overview; (**B**) enlarged view of the section enclosed by the red square in Fig. (**A**). Staining shows that the layers of tissue have degenerated into a strongly flexed form.

Massive pannus tissue was observed to cover the left atrial side of the prosthetic valve circumferentially, leaving a central pinhole with a diameter of 5 mm (**Fig. 1B**). We excised the pannus and prosthetic valve (**Fig. 2**) and, thereafter, implanted a 27-mm St. Jude Medical Epic bioprosthesis (St. Jude Medical, St. Paul, MN, USA) with pledgeted non-everting mattress sutures. Postoperatively, the patient recovered well and was discharged on postoperative day 83. The macroscopic and microscopic findings of the removed bioprosthesis revealed an accordion-like leaflet deformation on the ventricular side (**Figs. 2** and **3**), which shortened the leaflet and caused severe MR.

## DISCUSSION

In this report, we describe a case of severe bioprosthetic valve failure due to massive pannus formation successfully treated with redo MVR. Pannus formation is a well-recognized cause of bioprosthetic valve failure and is thought to be associated with chronic inflammatory changes. However, its precise etiology remains unclear. Previous studies have reported that pannus formation typically occurs around 5 years after prosthetic valve implantation.^3)^ Additionally, younger age and smaller prosthetic valve size have been suggested as risk factors for pannus development. Longitudinal imaging follow-up using computed tomography and transthoracic echocardiography has been shown to be beneficial in detecting early-stage pannus and monitoring its progression.^3)^

Jamieson et al. reported on the long-term performance of the Mosaic porcine bioprosthesis, highlighting its stable hemodynamics over a 12-year period and a low incidence of reintervention, primarily attributed to causes other than pannus, such as leaflet tear.^4)^ Their study, which found pannus in only 4 of 232 cases (including other reports in this paper, there are no cases of pannus), suggests that severe pannus formation, as seen in our case, is rare, thereby underlining the associated challenges and unique nature of our findings.

Bernard et al. also reported the long-term performance of the Epic bioprosthesis, highlighting its stable hemodynamics over a 5-year period and a low incidence of reintervention, primarily attributed to causes other than pannus, such as leaflet tear and endocarditis.^1)^ They found pannus formation in only 1 out of 1397 cases, indicating that severe pannus formation, as seen in our case, is exceedingly rare.

In our case, the pannus extended centrally from the sewing cuff on the left atrial side, without direct attachment to the leaflets. This suggests that the pannus may have originated from the sewing cuff, the sutures, or the pledgets on the atrial side. Considering the possibility of inflammation caused by the pledgets and subsequent pannus formation, we chose non-everting mattress sutures placing the pledgets on the left ventricular side. Additionally, to prevent any interference with the bioprosthetic valve on the left ventricular side, the posterior leaflet tissue was excised.

Importantly, surgical treatment allowed us to obtain valuable photographic and pathological findings of the failed bioprosthesis. Intraoperative findings indicated that navigating a transcatheter valve through the pinhole-sized opening would have been challenging. Additionally, pre-dilation with a balloon likely conferred a high risk of embolism. Moreover, there was a potential for incomplete expansion of the transcatheter valve post-implantation.

The previous study has compared the outcomes of two treatment options for mitral bioprosthetic valve failure: redo MVR and MVIV, and their short- to mid-term outcomes were found to be similar.^1)^ A systematic review comparing these two treatment options has found no statistically significant difference in the mortality, despite the higher incidence of major bleeding and arrhythmia in the redo MVR group.^5)^ They also noted that anatomical limitations might prevent transcatheter options from fully addressing the underlying pathology. The anatomical details, as well as the patient’s surgical risk, should be carefully considered in the decision-making of the reintervention.

## CONCLUSIONS

We have experienced a case of severe mitral bioprosthetic valve failure due to massive pannus formation that was successfully treated with redo surgical MVR. The intraoperative and pathological findings are invaluable for enhancing the safety and effectiveness of transcatheter interventions.

## DECLARATIONS

### Funding

None declared.

### Authors’ contributions

All authors have read and approved the manuscript.

RH: Writing-original draft, ST: Writing-review & editing, JI: Writing-review & editing, YO: Writing-review & editing, MT: Supervision, writing-review & editing.

### Availability of data and materials

Data are available on request to the corresponding author.

### Ethics approval and consent to participate

This work does not require ethical considerations or approval.

### Consent for publication

Informed consent was obtained from the patient.

### Competing interests

The authors have no conflicts of interest.
